# Development and validation of the core life activities scale

**DOI:** 10.3389/fpsyg.2024.1359276

**Published:** 2024-04-22

**Authors:** Surin Cho, Kiho Park, Dawoon Jung, Gaeun Son, Eunsil Cho, Kee-Hong Choi

**Affiliations:** ^1^School of Psychology, Korea University, Seoul, Republic of Korea; ^2^KU Mind Health Institute, Korea University, Seoul, Republic of Korea; ^3^Mindeep CBT Center, Seoul, Republic of Korea

**Keywords:** screening tests, core life activities, psychometrics, activities of daily life, activities of daily living, scale development, validation

## Abstract

Life activities profoundly influence well-being, mental health, and quality of life. The COVID-19 pandemic has heightened the importance of monitoring these activities for psychological and emotional health. However, existing measurement tools are limited, particularly for assessing psychological health. To address this gap, we developed and validated the Core Life Activities (CORE) scale, comprising five key factors (sleep, exercise, learning, diet, and social relationships) identified in neuroscience, cognitive psychology, and gerontology. In Study 1 (*n* = 1,137), exploratory and confirmatory factor analyses supported a single-factor structure with good model fit (χ^2^ = 6.377, *df* = 3, TLI = 0.992, CFI = 0.998, RMSEA = 0.031), demonstrating robust internal consistency (Cronbach’s alpha = 0.776) and test–retest reliability (intraclass correlation coefficient = 0.522, *p* < 0.001). The CORE exhibited significant convergent validity with mental health screening tools for depressive and anxiety disorders and suicidality. Study 2 (*n* = 684) confirmed a significant correlation between CORE and the World Health Organisation Quality of Life Brief Version, complementing the convergent validity found in Study 1. In addition, discriminant validity was confirmed by a non-significant correlation with the COVID-19 Preventive Behavior Scale. The findings establish the CORE as a reliable and valid tool, offering a simple yet comprehensive measure for assessing core life activities with potential applications in diverse environments.

## 1 Introduction

Life activities are essential elements that make up the daily routines necessary for the continuation of one’s life. Each day comprises 24 h of life activities, and when these days accumulate, they form an individual’s life. In other words, life activities are deeply intertwined with a person’s life. Diseases or injuries are often the first factors that impact life activities. Similarly, natural disasters, weather conditions, and events such as pandemics result in huge changes in daily activities. There have been significant changes in life activities following the COVID-19 pandemic. These changes encompass sleep, eating habits, exercise routines, education and learning environments, and how people interact with others (Dwyer et al., 2020; Bennett et al., 2021; Okabe-Miyamoto et al., 2021; Partinen, 2021; Pokhrel and Chhetri, 2021). Changes in life activities lead to discomfort and significantly impact an individual’s well-being and mental health (Caroppo et al., 2021). Challenges in carrying out daily life activities are linked to an increased likelihood of experiencing depression and anxiety (Judd et al., 2000; Olatunji et al., 2007), which can potentially undermine overall mental health and quality of life (Rapaport et al., 2005). Struggles in performing life activities can elevate suicidal ideation, and suicidal thoughts can potentially promote suicidal behavior (Xu et al., 2016; Khazem and Anestis, 2019). Indeed, life activities are closely tied to mental health, and the indirect and direct changes in life activities following events such as the COVID-19 pandemic have highlighted their significance. Considering the possibility of similar pandemics in future, it is crucial to acknowledge the need for adaptations and changes in life activities (Caroppo et al., 2021). Therefore, in anticipation of future pandemic situations, attention to life activities is vital for maintaining psychological health and well-being.

Daily activity is also strongly associated with a person’s overall quality of life, which means that measuring and monitoring daily activity levels can have a positive impact on a person’s quality of life (Edemekong et al., 2022). In behavioural activation therapy, one of the structured short-term treatments developed to treat depression, activity monitoring is included in all Behavioural Activation manuals, suggesting that activity monitoring is a fundamental part of behavioural activation. Studies have shown that daily activity monitoring reduces the frequency of problem behaviours and depressive symptoms and increases activity levels (Lee et al., 2016). In summary, as life activities are closely associated with variables such as depression and quality of life, monitoring life activities can be valuable in psychotherapy and counseling, helping reduce emotional issues and enhance treatment effectiveness. Monitoring changes in life activities is expected to enable the early detection of potential psychological difficulties, allowing for more effective interventions. Additionally, monitoring makes it possible to detect imbalances in an individual’s daily life and identify the personal and environmental factors contributing to them, thus facilitating more targeted interventions.

In daily life, individuals engage in diverse types and categories of life activities. However, core life activities are crucial to an individual’s mental health. Therefore, this study aims to develop and validate a tool for quickly measuring core life activities. Life activities are directly or indirectly related to an individual’s mental health (Jonsdottir et al., 2010; Xu et al., 2010), and as they encompass all activities that we perform in our daily lives, the categories are broad (Bieńkiewicz et al., 2014). Neuroscience, cognitive psychology, gerontology, and other fields have identified five key life activities related to maintaining brain health: social relationships, exercise, learning, diet, and sleep (Arden, 2014; Arden, 2023). In this study, these activities are considered as core life activities. As core life activities are significantly related to both brain health and an individual’s mental well-being, measuring and monitoring these activities can be a valuable tool for enhancing the effectiveness of psychological therapy and counseling. However, it is worth noting that tools for measuring core life activities are relatively scarce. While there are scales for measuring life activities, they may have limitations regarding applicability in psychological research or therapy, including their target population and the number of assessment items.

Activities of daily living (ADL) and a healthy lifestyle are essential to life activities. The American Psychological Association (Vanden Bos, 2007) defines ADL as “activities essential to an individual’s care.” It includes basic self-care activities such as personal grooming, dressing, toileting, mobility, and eating. In other words, ADL reflect an individual’s ability to function independently and how often they can do so (Mlinac and Feng, 2016). Most prior studies on ADL have targeted older adults or individuals with specific medical conditions or disabilities. For instance, the Modified Barthel Index (MBI; Shah et al., 1989, 1998) includes items such as “The patient is unable to climb stairs,” which may not be suitable for the general population. Additionally, the Klein-Bell ADL Scale (Klein and Bell, 1982) has many items (170), which may limit its usability and practicality. The Frenchay Activities Index (Holbrook and Skilbeck, 1983) includes gardening (weeding, pruning) and painting, which are common in specific cultures, making it less applicable in different cultural contexts.

Lifestyle is another crucial concept related to life activities, defined by the APA (2007) as “the typical way of life or manner of living that is characteristic of an individual or group, as expressed by behaviors, attitudes, interests, and other factors.” It encompasses an individual’s daily activities, behaviors, attitudes, interests, and more. The majority of prior studies have addressed lifestyle from a health perspective (Fortier, 2015). Indeed, the existing tools developed to assess lifestyle primarily consist of items that reflect a healthy way of life. For instance, the Health Enhancement Lifestyle Profile (Hwang, 2010) includes items such as “Check your health condition at home,” “Participate in health information sessions,” and “Watch TV programs or listen to radio programs on health.” The Health Promoting Lifestyle Profile-II (Walker et al., 1995) comprises specific items related to health promotion, such as “Attend educational programs on personal health care,” “Ask for information from health professionals about how to take good care of myself,” and “Inspect my body at least monthly for physical changes/danger signs.” The Healthy Lifestyle Scale for University Students (Wang et al., 2012) and the Healthy Lifestyle and Personal Control Questionnaire (Darviri et al., 2014) include items that may not align with modern lifestyles, for example, “Eat breakfast daily,” “Read or use the computer continuously for more than 1 h,” and “Listen continuously to headphones for more than 30 min.” Additionally, the Healthy Lifestyle Scale for University Students includes culturally specific items, such as “I use dairy products such as milk, yogurt, and cheese two or more times every day,” which may not be appropriate for many East Asians with lactose intolerance. In summary, existing scales for measuring life activities have limitations, as they may have restricted applicability, contain excessive items, include activities that are not considered core activities, or feature outdated or culturally specific content. Furthermore, these scales often focus on physical health and may not be suitable for assessing mental health. Therefore, these tools may not directly capture core life activities, as they may contain content that needs to be explicitly aligned with the core activities.

Furthermore, a tool for measuring life activities should be user-friendly (Klimczuk, 2016). Using a self-report format can save costs and enhance usability (Cress et al., 1995). Especially, when targeting patients, it is crucial to design a measurement tool that allows respondents to answer quickly (Sadura et al., 1992). Considering these factors, there is a need for a tool that respondents can complete quickly without being constrained by cultural, temporal, racial, or other factors. Furthermore, the questionnaire’s content should balance being sufficiently detailed as well as broad while reflecting the essential aspects of daily life. Additionally, the tool must be applicable within the realm of mental health. The objective of this study is to develop a scale that can measure core daily activities, addressing the limitations of existing scales. To achieve this goal, we developed and validated the self-report Core Life Activities Scale (CORE) that measures five fundamental elements (sleep, diet, exercise, social relationships, and learning) among the daily activities essential to human life.

## 2 Study 1

### 2.1 Materials and methods

#### 2.1.1 Participants

Study 1 used data from two different surveys collected at different times. Participants were recruited nationwide through recruitment advertisements on online survey agencies, and data collection at both time points was done through online surveys. Participants were randomly selected based on gender, age, education and regional distribution of the total population of South Korea. To ensure the quality of the surveys, real names were verified to prevent the same person from participating in multiple surveys. A total of 1,137 participants were recruited for the first survey (Time1) of Study 1. To measure test–retest reliability, 910 of the 1,137 participants from the first survey (Time1) completed the CORE at the second survey (Time2), eight weeks later. The inclusion criteria were as follows: (1) aged 18 and above and (2) able to easily read Korean. The exclusion criterion was providing inappropriate responses. Participation was voluntary, and all individuals provided written informed consent. The study received approval from the Institutional Review Board of Korea University (KUIRB-2021-0013-02). Relevant detailed demographic information is provided in Table 1.

**Table 1:** Sample demographics (study 1 and study 2).

		Total sample (*N* = 1,137)	Total sample (*N* = 910)	Total sample (*N* = 684)
		Study 1 (Time 1)	Study 1 (Time 2)	Study 2
Sex	
Male		576 (50.7%)	458 (50.3%)	353 (51.6%)
Female	561 (49.3%)	452 (49.7%)	331 (48.4%)
Age
20s	199 (17.5%)	134 (14.7%)	94 (13.7%)
30s	215 (18.9%)	165 (18.1%)	107 (15.6%)
40s	255 (22.4%)	208 (22.9%)	160 (23.4%)
50s	269 (23.7%)	231 (25.4%)	188 (27.5%)
60s	199 (17.5%)	172 (18.9%)	135 (19.7%)

#### 2.1.2 Measures

##### 2.1.2.1 Core life activities scale

The CORE is a tool developed to assess the level of daily activities in a Korean sample. This self-report scale consists of five items rated on a 5-point Likert scale (1 = never, 2 = rarely, 3 = sometimes, 4 = usually, 5 = always). The CORE consists of eating habits, sleep pattern, exercising, learning about new things, and social relationships.

##### 2.1.2.2 Mental health screening tool for depressive disorders

The Mental Health Screening Tool for Depressive Disorders (MHS: D) is a tool to assess the level of depression in a Korean sample. This self-report instrument consists of 12 items rated on a 5-point Likert scale (0 = never; 4 = always). The MHS:D demonstrated significant and high levels of validity, as indicated by substantial correlations with the Beck Depression Inventory-II, Patient Health Questionnaire-9, and Center for Epidemiologic Studies Depression Scale. The Cronbach’s alpha coefficient for internal consistency was 0.95 (Park et al., 2022).

##### 2.1.2.3 Mental health screening tool for anxiety disorders

The Mental Health Screening Tool for Anxiety Disorders (MHS:A) is a tool to assess the level of anxiety in a Korean sample. This self-report instrument consists of 11 items rated on a 5-point Likert scale (0 = never, 4 = always). The MHS:A demonstrated significant and high levels of validity, as indicated by substantial correlations with the Beck Anxiety Inventory, Penn State Worry Questionnaire, and the Generalized Anxiety Disorder-7. The Cronbach’s alpha coefficient for internal consistency was 0.97 (Kim et al., 2021).

##### 2.1.2.4 The ultra brief checklist for suicidality

The Ultra Brief Checklist for Suicidality (UBCS) is designed to assess suicide risk in minimal time. It comprises four self-report items and is available via paper or the Internet. The items are rated on a 5-point Likert scale (0 = never; 4 = always). It showed robust reliability and validity among a Korean sample. Cronbach’s alpha coefficient for internal consistency was 0.82 (Yoon et al., 2018, 2020).

#### 2.1.3 Development and validation of the CORE

##### 2.1.3.1 Item generation

The researchers prepared a total of five question pools, consisting of one question representing each of the five core life activity categories. Afterwards, an expert group, including a professor in clinical psychology, five clinical psychologists, and three students in a clinical psychology doctoral program, determined whether the questions represented the level of core life activities. Consequently, it was agreed that all five questions reflected the concepts intended to be measured in this study. The CORE’s five questions ask respondents about daily activities “over the past week.” The relevant questions can be found in Table 2. All questions are responded to on a 5-point Likert scale, where higher scores indicate a higher level of core life activity.

**Table 2:** Descriptive characteristics and internal consistency of the CORE (study1).

	CORE		
	Mean (SD)	*r _tot_*	*α* if item deleted
1. Did you get enough sleep in the past week?	3.14 (0.96)	0.514	0.746
2. Did you eat a regular and balanced diet over the past week?	3.25 (0.95)	0.614	0.714
3. Have you been physically active enough in the past week?	2.92 (1.05)	0.614	0.712
4. Did you spend time, including phone calls, video calls, texts, emails, and so on, with family, friends, and close acquaintances over the past week?	3.44 (0.94)	0.509	0.748
5. Did you spend the last week learning something new, even if it was trivial?	2.75 (1.10)	0.506	0.752

##### 2.1.3.2 Procedure

Sample data used for statistical analysis were collected in May and July 2020. Participants completed the surveys online. For descriptive statistics, internal consistency reliability, test–retest reliability, exploratory factor analysis (EFA), and convergent validity were calculated, and SPSS Statistics 25 (IBM Corp., Armonk, NY, United States) was used. Confirmatory factor analysis was conducted using the R statistical program (version 3.5.0) with the “lavaan” package (Rosseel, 2012).

### 2.2 Results

#### 2.2.1 Construct validity

##### 2.2.1.1 Exploratory factor analysis

Construct validity was assessed using EFA to determine the core components of the five-item questionnaire. The Kaiser-Meyer-Olkin measure was 0.782, and Barlett’s test of sphericity showed a statistically significant level (χ^2^ = 1484.312***, *df* = 10, *p* < 0.0001), indicating the collected data were appropriate for factor analysis (Tabachnick et al., 2013). EFA revealed that a single factor explained 53.113% of the total variance of the construct.

##### 2.2.1.2 Confirmatory factor analysis

**Figure 1:**
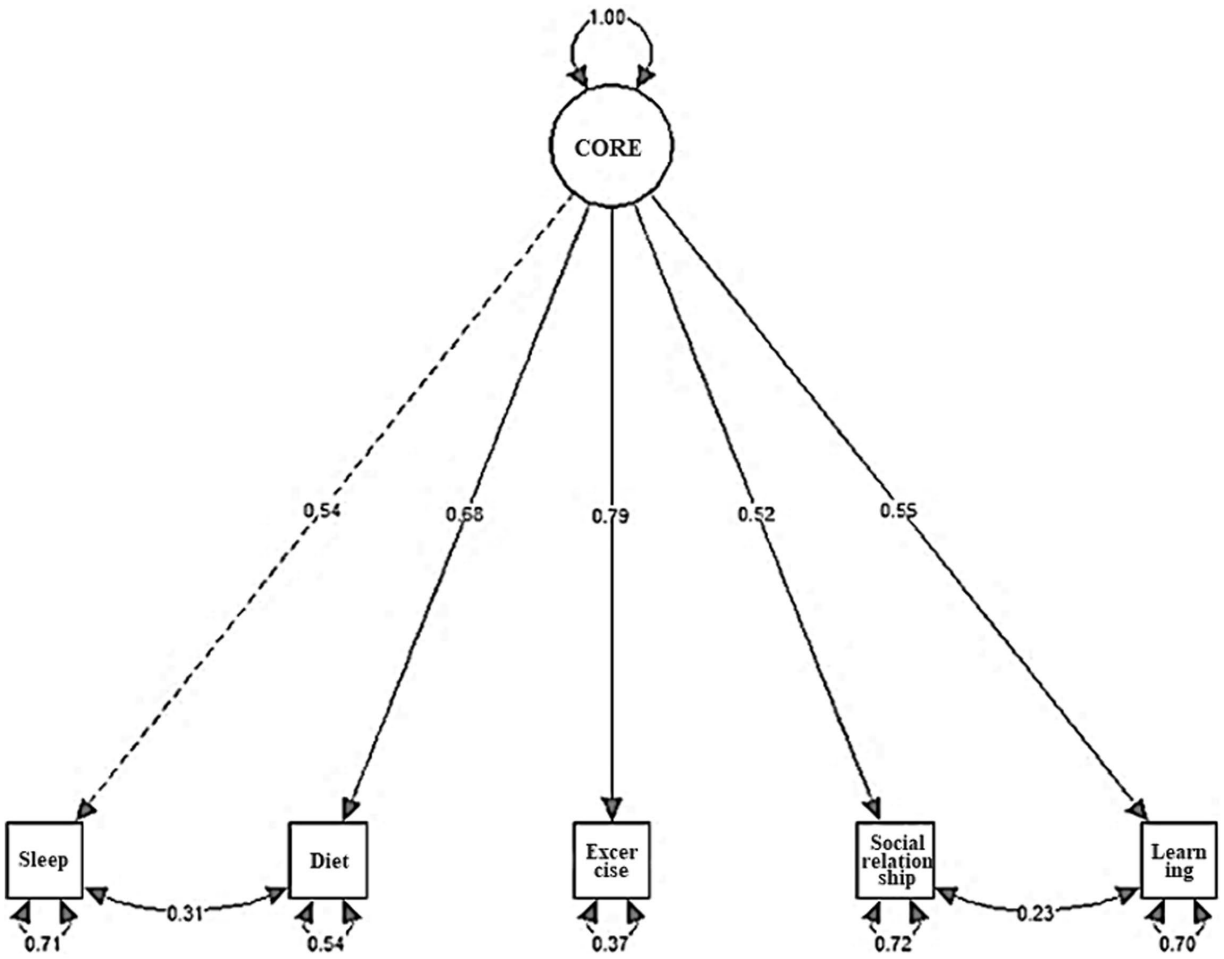
Results of the confirmatory factor analysis of the CORE (study 1).

The analysis revealed that the fit index of the one-factor model was χ^2^ = 120.434, *df* = 5, TLI = 0.844, CFI = 0.922, RMSEA = 0.142 (Table 3). On examining the modification index, it was found that the model fit for the CORE improved when the correlated residuals of items 1 and 2 and items 4 and 5 were included. The modification index of the covariance of the error term of question 1 (Did you get enough sleep in the past week?) and question 2 (Did you eat a regular and balanced diet over the past week?) was large. The two items were judged to be similar in that they were basic needs, and thus, the error terms of the two items were linked. Next, the modification index of the covariance of the error term was large for question 4 (Did you spend time, including phone calls, video calls, texts, emails, and so on, with family, friends, and close acquaintances over the past week?) and question 5 (Did you spend the last week learning something new, even if it was trivial?). These items were judged to be similar in that social interactions and learning are deeply related (Okita, 2012). Therefore, the error terms of the two items were linked. As a result of re-analysis, the CFI value rose to 0.998, the TLI value to 0.992, and the RMSEA to 0.031. Therefore, the model to which the modification index was applied was selected as a suitable model. The standardized coefficient estimates for the one-factor model using the modified indices are presented in Table 3 and Figure 1.

**Table 3:** Goodness-of-fit indices for the one-factor model of the CORE (study 1).

	χ^2^	*df*	p	TLI	CFI	RMSEA			SRMR
						Value	Lower bound	Upper bound	
Before	120.434	5	0.000	0.844	0.922	0.142	0.121	0.165	0.053
After	6.377	3	0.000	0.992	0.998	0.031	0.000	0.066	0.011

*N* = 1,137, TLI, Tucker-Lewis Index, CFI, Comparative Fit Index, RMSEA, Root Mean. Square Error of Approximation, SRMR, Standardized Root Mean Square Residual.

#### 2.2.2 Internal consistency

The Cronbach’s alpha coefficient for the CORE was 0.776, indicating a high level of internal consistency. All five items contributed to improving internal consistency; none needed to be excluded. Table 2 reports the Cronbach’s alpha coefficients when individual items were removed, along with the correlation coefficients between each item and the total score.

#### 2.2.3 Test–retest reliability

Test–retest reliability was analyzed by examining the correlation between the results of the CORE at Time 1 and Time 2. The test–retest reliability was significant (Intraclass coefficient = 0.522), with a 95% confidence interval from 0.473 to 0.568, *F* (910) = 3.204, *p* < 0.001, indicating good reliability (Fleiss, 1986).

When analysed stratified by gender, both females and males showed significant correlations in test–retest reliability for CORE scores. For males, the test–retest reliability was significant (Intraclass coefficient = 0.496), with a 95% confidence interval from 0.424 to 0.562, *F* (459) = 2.987, *p* < 0.001, indicating good reliability (Fleiss, 1986). For women, the test–retest reliability was significant (Intraclass coefficient = 0.547), with a 95% confidence interval from 0.479 to 0.608, *F* (451) = 3.429, *p* < 0.001, indicating good reliability. Females demonstrated higher levels of test–retest reliability than males, which was higher than the test–retest reliability of all participants.

When analysed stratified by age, test–retest reliability was demonstrated for all age groups. For those in their 20s, the test–retest reliability was significant (Intraclass coefficient = 0.345), with a 95% confidence interval from 0.189 to 0.485, *F* (136) = 2.055, *p* < 0.001. For those in their 30s, the test–retest reliability was significant (Intraclass coefficient = 0. 457), with a 95% confidence interval from 0.326 to 0.570, *F* (163) = 2.676, p < 0.001, indicating good reliability. For those in their 40s, the test–retest reliability was significant (Intraclass coefficient = 0.547), with a 95% confidence interval from 0.445 to 0.636, *F* (208) = 3.453, *p* < 001, indicating good reliability. In the 50s, the test–retest reliability was significant (Intraclass coefficient = 0.513), with a 95% confidence interval from 0.409 to 0.603, *F* (231) = 3.187, p < 0.001, indicating good reliability. In the 60s, the test–retest reliability was significant (Intraclass coefficient = 0. 662), with a 95% confidence interval from 0.569 to 0.738, *F* (172) = 4.918, p < 0.001, indicating good reliability. The 60s had the highest level of test–retest reliability, which was higher than the test–retest reliability of all participants.

#### 2.2.4 Convergent validity

To validate the convergent validity of the CORE, correlation analyses were performed with the MHS:D, MHS:A and UBCS. The total CORE score was significantly negatively correlated with the total MHS:D (*r* = −0.312, *p* < 0.01), MHS:A (*r* = −0.311, *p* < 0.01) and UBCS (*r* = −0.088, *p* < 0.01) scores.

When stratified by gender, men’s CORE scores were significantly negatively correlated with total MHS:D (*r* = −250, *p* < 0.01), MHS:A (*r* = −0.258, *p* < 0.01) and UBCS (*r* = −0.042, *p* < 0.01) scores. Women’s CORE scores were significantly negatively correlated with total MHS:D (*r* = −0.369, *p* < 0.01), MHS:A (*r* = −0.361, *p* < 0.01) and UBCS (*r* = −0.134, *p* < 0.01) scores. MHS:D, MHS:A and UBCS all showed higher negative correlations with CORE in women than in men.

When stratified by age, CORE scores in the 20s were significantly negatively correlated with total MHS:D (*r* = −0.304, *p* < 0.01) and MHS:A (*r* = −0.251, *p* < 0.01) scores, while the correlation with total UBCS scores was not significant. CORE scores in the 30s were not significantly correlated with total MHS:D, MHS:A and UBCS scores; CORE scores in the 40s were significantly correlated with total MHS:D (*r* = −0.406, *p* < 0.01), MHS:A (*r* = −0.379, *p* < 0.01) and UBCS (*r* = −0.132, *p* < 0.01) scores. CORE scores in the 50s were significantly negatively correlated with total MHS:D (*r* = −0.295, *p* < 0.01) and MHS:A (*r* = −0.322, *p* < 0.01) scores, while the correlation with total UBCS scores was not significant. CORE scores in the 60s were significantly negatively correlated with total MHS:D (*r* = −0.493, *p* < 0.01), MHS:A (*r* = −0.479, *p* < 0.01) and UBCS (*r* = −0.285, *p* < 0.01) scores. MHS:D, MHS:A and UBCS all had the highest negative correlations with CORE in the 60s.

Table 4 shows the detailed correlation coefficients.

**Table 4:** Correlation coefficients of the CORE total score with other scales (study1).

	CORE							
		Sex		Age				
	Total sample (*N* = 1,137)	Male (*N* = 576)	Female (*N* = 561)	20s (*N* = 199)	30s (*N* = 215)	40s (*N* = 255)	50s (*N* = 269)	60s (*N* = 199)
MHS:D	−0.312^**^	−0.250^**^	−0.369^**^	−0.304^**^	−0.075	−0.406^**^	−0.295^**^	−0.493^**^
MHS:A	−0.311^**^	−0.258**	−0.361^**^	−0.251^**^	−0.089	−0.379^**^	−0.322^**^	−0.479^**^
UBCS	−0.088^**^	−0.042^**^	−0.134^**^	−0.104	0.085	−0.132^**^	−0.062	−0.285^**^

^**^p < 0.01, MHS:D, Mental Health Screening tool for Depressive disorders, MHS:A, Mental Health Screening tool for Anxiety disorders, UBCS, Ultra Brief Checklist for Suicidality, CORE, Core Life Activities Scale.

## 3 Study 2

In Study 2, to supplement the validity of the CORE confirmed in Study 1, we examined convergent validity with scales that are believed to be more conceptually related to the CORE, and additionally examined discriminant validity with scales that are believed to be semantically dissimilar to the CORE.

### 3.1 Materials and methods

#### 3.1.1 Participants

In Study 2, 684 participants were recruited to complete the survey to complement the convergent validity of the CORE. Participants were recruited from across the country through recruitment advertisements on online survey agencies and were administered an online survey. Participants were randomly selected based on gender, age, education and regional distribution of the total population of South Korea. To ensure the quality of the survey, real names were verified to prevent the same person from participating in multiple surveys. The inclusion criteria were as follows: (1) aged 18 and above (2) able to easily read Korean. The exclusion criterion was providing inappropriate responses. Participation was voluntary, and all individuals provided written informed consent. The study received approval from the Institutional Review Board of Korea University (KUIRB-2021-0013-02). Relevant detailed demographic information is provided in Table 1.

#### 3.1.2 Measures

##### 3.1.2.1 Core life activities scale

This tool is identical to the one utilized in Study 1.

##### 3.1.2.2 World Health Organization quality of life brief version

The World Health Organization Quality of Life Brief Version (WHOQOL-BREF) was designed to provide a precise and convenient measure of quality of life. This self-reported instrument consists of 26 items rated on a 5-point Likert scale (1 = not at all; 5 = most of the time). In this study, the Korean version of the test was used (Min et al., 2000), demonstrating satisfactory internal consistency (Cronbach’s alpha = 0.898).

##### 3.1.2.3 COVID-19 preventive behavior scale

The COVID-19 Preventive Behavior Scale (CPBS) was employed to assess divergent validity. This instrument was developed based on the connection between psychological factors and behaviors associated with infectious diseases. This self-reported instrument consists of eight items rated on a 5-point Likert scale (0 = never; 4 = most of the time). The internal consistency, as measured by Cronbach’s alpha coefficient, was 0.88 (Bahk et al., 2020).

#### 3.1.3 Procedure

Sample data used for statistical analysis were collected in December 2020. Participants completed the survey online. Convergent and divergent validity were assessed using SPSS Statistics 25 (IBM Corp., Armonk, NY, United States).

### 3.2 Results

#### 3.2.1 Convergent validity

To verify the convergent validity of the CORE, correlation analysis was performed with the WHOQOL-BREF. The total score of the CORE exhibited a significant correlation with the total score of the WHOQOL-BREF (*r* = 0.571, *p* < 0.01). Additionally, there was a significant correlation between the CORE and all domains of the WHOQOL-BREF (physical health, *r* = 0.513; psychological health, *r* = 0.476; social relationships, *r* = 0.454; environmental health, *r* = 0.498; *p* < 0.01). Table 5 contains detailed correlation coefficients.

**Table 5:** Correlation coefficients of the CORE total score with other scales (study2).

	CORE							
	Sex				Age			
	Total sample (*N* = 684)	Male (*N* = 353)	Female (*N* = 331)	20s (*N* = 94)	30s (*N* = 107)	40s (*N* = 160)	50s (*N* = 188)	60s (*N* = 135)
WHOQOL-BREF Total	0.571 ^**^	0.536^**^	0.611^**^	0.400^**^	0.567^**^	0.488^**^	0.627^**^	0.672^**^
WHOQOL-BREF Physical health	0.513^**^	0.458^**^	0.566^**^	0.405^**^	0.507^**^	0.434^**^	0.545^**^	0.621^**^
WHOQOL-BREF Psychological health	0.476^**^	0.464**	0.487^**^	0.245^**^	0.439^**^	0.393^**^	0.523^**^	0.605^**^
WHOQOL-BREF Social relationships	0.454^**^	0.449^**^	0.467^**^	0.362^**^	0.497^**^	0.394^**^	0.491^**^	0.513^**^
WHOQOL-BREF Environmental health	0.498^**^	0.459^**^	0.548^**^	0.373^**^	0.489^**^	0.419^**^	0.554^**^	0.579^**^
CPBS	0.024	0.042	0.095	−0.050	0.202	0.130	−0.118	−0.061

*N* = 684, ^**^p < 0.01, WHOQOL-BREF: abbreviated World Health Organization Quality of Life Scale, CPBS: COVID-19 Preventive Behavior Scale.

When stratified by gender, for both women and men, there was a significant correlation between CORE total scores and WHOQOL-BREF total scores and all domains of the WHOQOL-BREF. Men’s total score on the CORE was significantly correlated with their total score on the WHOQOL-BREF (*r* = 0.536, *p* < 0.01). There were also significant correlations between the CORE and all subdomains of the WHOQOL-BREF (physical health, *r* = 0.458; psychological health, r = 0.464; social relationships, *r* = 0.449; environmental health, *r* = 0.459; *p* < 0.01). The total CORE score in women was significantly correlated with the total WHOQOL-BREF score (*r* = 0.611, *p* < 0.01). There were also significant correlations between the CORE and all subdomains of the WHOQOL-BREF (physical health, *r* = 0.566; psychological health, *r* = 0.487; social relationships, *r* = 0.467; environmental health, *r* = 0.548; *p* < 0.01). Women had higher correlations with the CORE than men for both the total score and the subdomains of the WHOQOL-BREF.

When stratified by age group, there was a significant correlation between CORE total scores and WHOQOL-BREF total scores and all domains of the WHOQOL-BREF across all age groups. CORE total scores in the 20s were significantly correlated with WHOQOL-BREF total scores (*r* = 0.400, *p* < 0.01) and all subdomains of the WHOQOL-BREF (physical health, *r* = 0.405; psychological health, *r* = 0.245; social relationships, *r* = 0.362; environmental health, *r* = 0.373; *p* < 0.01). In addition, the total CORE score in the 30s was significantly correlated with the total WHOQOL-BREF score (*r* = 0.567, *p* < 0.01) and all subdomains of the WHOQOL-BREF (physical health, *r* = 0.507; psychological health, *r* = 0.439; social relationships, r = 0.497; environmental health, *r* = 0.489; *p* < 0.01). The results were similar for those in their 40s as for those in their 20s and 30s, with the total score on the CORE significantly correlating with the total score on the WHOQOL-BREF (*r* = 0.488, *p* < 0.01). There were also significant correlations between the CORE and all subdomains of the WHOQOL-BREF (physical health, *r* = 0.434; psychological health, *r* = 0.393; social relationships, *r* = 0.394; environmental health, *r* = 0.419; *p* < 0.01). In addition, CORE correlated significantly with the total score and all subdomains of the WHOQOL-BREF in the 50s and 60s, as it did in other age groups. The total score of the CORE in the 50s was significantly correlated with the total score of the WHOQOL-BREF (*r* = 0.627, *p* < 0.01) and all subdomains of the WHOQOL-BREF (physical health, *r* = 0.545; psychological health, *r* = 0.523; social relationships, *r* = 0.491; environmental health, *r* = 0.554; *p* < 0.01). The total score of the CORE in the 60s was significantly correlated with the total score of the WHOQOL-BREF (*r* = 0.672, *p* < 0.01) and all subdomains of the WHOQOL-BREF (physical health, *r* = 0.621; psychological health, *r* = 0.605; social relationships, *r* = 0.513; environmental health, *r* = 0.579; *p* < 0.01). Correlations between the total score of the CORE and both the total score and subdomains of the WHOQOL-BREF were highest in the 60s.

Table 5 shows the detailed correlation coefficients.

#### 3.2.2 Divergent validity

There was no significant correlation between the total scores of the CORE and CPBS (*r* = 0.024, *p* = 0.536), confirming divergent validity. When stratified by gender, there was no significant correlation between CORE total scores and CPBS scores for either men or women (men: *r* = 0.042, *p* = 0.627; women: *r* = 0.095, *p* = 0.073). When stratified by age, there was no significant correlation between CORE total scores and CPBS scores across all age groups (20s: *r* = −0.050, *p* = 0.364; 30s: *r* = 0.202, *p* = 0.051; 40s: *r* = 0.130, *p* = 0.183; 50s: *r* = −0.118, *p* = 0.138; 60s: *r* = −0.061, *p* = 0.404). Table 5 shows the detailed correlation coefficients.

## 4 Discussion

The purpose of this two-part study was to develop and validate a scale to assess the core activities of daily living. We developed a five-item scale, titled the CORE, rated on a 5-point Likert scale. In Study 1, CORE items were developed, and a one-factor structure comprising five items was validated through EFA and confirmatory factor analysis. Reliability was ensured by assessing internal consistency and through a test–retest conducted at an eight-week interval.

The ICC for test–retest reliability is between 0.4 and 0.75, indicating a good level of reliability (Fleiss, 1986). In this study, test–retest reliability was good for all participants, and when stratified by gender, both men and women had good test–retest reliability, with women having higher ICC values than men and all participants. This may be because women tend to be more aware and understanding of their psychological state and emotional changes than men, and therefore have more consistent results when retested (Brody, 1993). Furthermore, when test–retest reliability was stratified by age group, test–retest reliability was significant for all age groups from the 20s to the 60s. All age groups except the 20s had good test–retest reliability, and the 60s had the highest test–retest reliability of all age groups, which may be due to more life experience, mature judgement and more consistent results with age (Ardelt, 2010).

In Study 1, CORE also showed significant correlations with measures of depression, anxiety, and suicide, confirming convergent validity. Other studies have also found significant associations between activities of daily living and depression, particularly in older adults, patients with Parkinson’s disease, and stroke patients (Chemerinski et al., 2001; Lawrence et al., 2014; Mohamadzadeh et al., 2020). In addition, patients with depression show symptoms of sleep disturbance, decreased or increased appetite, decreased physical activity, social isolation and withdrawal, and and withdrawal from learning in areas related to daily living (Berridge and Kringelbach, 2008; Stafford et al., 2011; Lai et al., 2014; Kandola et al., 2019; Steiger and Pawlowski, 2019). These findings are consistent with the significant association between the CORE and depression found in this study. In behavioral activation, monitoring of daily activities is mandatory for patients with depression and is used as a clue to identify the direction of therapeutic intervention for them (Spates et al., 2006). The CORE appears to be able to efficiently provide clues for treatment for individuals with depression. Alternatively, even without a diagnosis of depression, the CORE can be useful in preventing depression or managing emotions by observing changes in the CORE and depression levels.

The significant association between anxiety and activities of daily living found in this study has also been investigated in other studies. For example, correlations between anxiety and daily activities have been found in stroke patients, older adults, and patients with Parkinson’s disease (Dissanayaka et al., 2010; Kempen et al., 2012; Tang et al., 2013). While anxiety can cause disruption of daily rhythms such as sleep, anxiety can also be caused by disruption of daily activities (Alvaro et al., 2013). It has also been suggested that individuals experiencing high levels of anxiety may seek protection from anxiety symptoms and related disorders through daily activities, such as physical activity (McDowell et al., 2019). Similarly, other studies have found a significant association between anxiety and daily activities, which is consistent with the findings of this study. This suggests that the CORE could be used as a tool to identify factors that may contribute to anxiety, and to examine daily activities to reduce or prevent anxiety disorders.

As with depression and anxiety, suicide is significantly associated with activities of daily living. This was confirmed in the present study, and other studies have also reported significant associations between levels of activities of daily living and suicidal ideation in populations such as older adults and stroke patients (Fuller-Thomson et al., 2012; Zhao et al., 2020). Reduced activity levels can increase depression, and lifestyle can influence depression and anxiety (Ferster, 1973; Noordsy, 2019). It has also been reported that low activity levels are significantly negatively correlated with mental health and quality of life (Wada et al., 2004). This may have a compounding negative effect on suicide (Seo et al., 2011). In addition, signs of suicide are associated with changes in daily routines, such as sudden changes in sleep patterns or social withdrawal (Rudd et al., 2006). The association between suicide and daily activities found in this study is consistent with other studies pointing in the same direction. While existing suicide screening tools can be used to assess suicide risk, the CORE is expected to be useful as a tool to identify drastic changes in daily activities in the early stages of suicide risk.

The correlations between CORE and depression, anxiety and suicidality found in Study 1 were significant, but not at high levels. There is no doubt that the relationships between daily functioning and depression, anxiety and suicidality are significant and interrelated. However, to better reflect the concept of convergent validity, it was necessary to look at correlations with other scales that could be considered to measure the same thing as CORE. In Study 2, we supplemented the convergent validity of the CORE identified in Study 1 with correlations with the WHOQOL-BREF, which is considered to be semantically related to the CORE.

As anticipated, a significant correlation was found between the WHOQOL-BREF and CORE. According to existing literature, the level of daily activity and quality of life are closely related in older adults, breast cancer patients, people with dementia, and stroke patients (Rietman et al., 2003; Haghgoo et al., 2013; Giebel et al., 2015; Sováriová Soósová, 2016). This evidence is largely based on studies involving older adults or individuals with physical illnesses. Nevertheless, the present study confirmed that there is a deep relationship between the level of daily activity and the level of quality of life in adult men and women of various age groups. In addition, the CORE showed a significant correlation not only with the overall quality of life, but also with all subdomains of the WHOQOL-BREF: physical health, psychological health, social relationships, and environmental health. This suggests that the CORE sufficiently reflects key aspects of life.

This study also analysed the relationship between WHOQOL-BREF and CORE stratified by gender and age group. The results showed significant correlations between WHOQOL-BREF and CORE in all sex and age groups, and between CORE and all subdomains of WHOQOL-BREF (physical health, psychological health, social relationships and environmental health). When comparing men and women, women had higher correlations than men in all domains, even higher than when looking at all participants. When comparing age groups, those in their 20s had the lowest correlations and those in their 60s had the highest correlations in all domains. These results were similar to those for test–retest reliability. In Study 2 we also looked at correlations with the CPBS, which is thought to measure concepts unrelated to the CORE, and found no significant correlations, providing discriminant validity.

Although this study confirmed the CORE’s factor structure, internal consistency, test–retest reliability, convergent validity, and discriminant validity, there are certain limitations to the study and its findings. First, when developing an index, it is generally suggested to create an item pool that is at least twice the final number of items (DeVellis and Thorpe, 2021). In this study, although the main factors of core activities were identified through literature research and expert meetings and reviews were conducted to determine whether the questions based on those factors represented core daily activities, the initial item pool was created with a small number of five questions. Even with these limitations, the initial item pool of five questions in the CORE demonstrated the suitability of a one-factor structure, and studies 1 and 2 substantiated a high level of reliability and validity for these items. However, it is necessary to supplement the present findings by preparing a broader item pool in future research.

Secondly, the time interval for test–retest reliability was rather long, which is a limitation. The time interval for test–retest reliability should be long enough to ensure that responses are not recalled from memory, but at the same time short enough to ensure that participants’ circumstances and conditions do not change (Lee, 2021). A commonly recommended time interval is 2 weeks (Streiner et al., 2015). The test–retest reliability interval for CORE was 8 weeks, which may have been too long. This may be the reason why the test–retest reliability in this study was only good, not excellent. In future studies, it is necessary to improve the reliability by shortening the test–retest interval.

Finally, this study was only conducted in the Korean cultural context. It is unclear whether the structure of the CORE would be different in other cultures. Therefore, it is important for future research to investigate the reliability and validity of the instrument in different cultural contexts.

In conclusion, this study developed and validated the CORE to assess core life activities. The five items of the CORE (sleep, diet, exercise, social relationships, and learning) yielded a one-factor structure, and the scale demonstrated robust internal consistency, test–retest reliability, and convergent and discriminant validity. Therefore, the CORE exhibits reliability and validity as a tool for assessing core activity levels.

## Data availability statement

The original contributions presented in the study are included in the article/supplementary material, further inquiries can be directed to the corresponding author.

## Ethics statement

The studies involving humans were approved by The Institutional Review Board of Korea University (KUIRB-2021-0013-02). The studies were conducted in accordance with the local legislation and institutional requirements. The participants provided their written informed consent to participate in this study.

## Author contributions

SC: Conceptualization, Writing – original draft, Writing – review & editing. KP: Formal analysis, Methodology, Writing – original draft. DJ: Methodology, Writing – original draft. GS: Writing – original draft, Writing – review & editing. EC: Writing – review & editing. K-HC: Resources, Supervision, Writing – original draft, Writing – review & editing.
